# Correction: Smith et al. MCAK Inhibitors Induce Aneuploidy in Triple-Negative Breast Cancer Models. *Cancers* 2023, *15*, 3309

**DOI:** 10.3390/cancers16020293

**Published:** 2024-01-10

**Authors:** John C. Smith, Stefan Husted, Jay Pilrose, Stephanie C. Ems-McClung, Jane R. Stout, Richard L. Carpenter, Claire E. Walczak

**Affiliations:** 1 Medical Sciences, Indiana School of Medicine—Bloomington, Bloomington, IN 47405, USA; jcs12@iu.edu (J.C.S.); scems@indiana.edu (S.C.E.-M.); janstout@indiana.edu (J.R.S.); richcarp@indiana.edu (R.L.C.); 2 LabCorp Drug Development Indianapolis, Indianapolis, IN 46214, USA; 3 Catalent Pharma Solutions Bloomington, Bloomington, IN 47403, USA

The authors alerted the Editorial Office of the mistake on 5 August 2023 and the final documents were sent for evaluation on 12 December 2023. In the original publication [1], there is a mistake in Figure 3F of the chemical structure for compound 2021-4 C4 (68806609). The six-membered ring at the bottom with the NH group should be a seven-membered ring. The corrected Figure 3 is included below in which the chemical structure was redrawn with ChemDraw rather than with Biorender. This change does not affect any data or scientific conclusions in the paper. We apologize for any inconvenience that this has caused. This correction was approved by the Academic Editor. The original publication has also been updated.

## Figures and Tables

**Figure 3:**
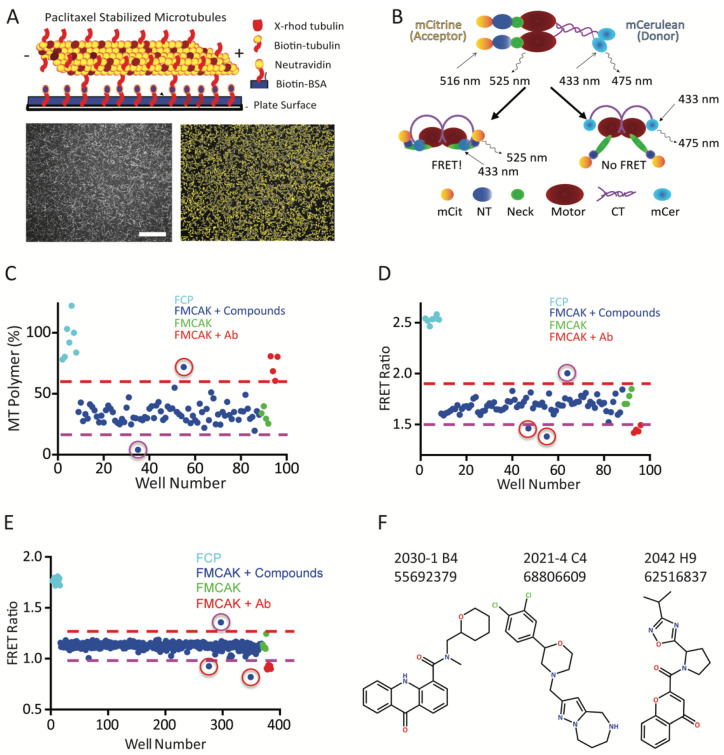
Identification of putative MCAK inhibitors. (**A**) Cartoon schematic and representative images of MT depolymerization assay. Lower left shows a representative image of the FCP control condition. Lower right shows the same image with the MTs identified by the FIJI algorithm outlined in yellow. Scale bar = 5 µm. (**B**) Schematic of MCAK FRET construct in which mCitrine (mCit) is fused to the N-terminus (NT) and mCerulean (mCer) is fused to the C-terminus (CT) of MCAK. Dimeric MCAK is in the closed active state in which FRET will occur (bottom left) or the open inactive state in which the FRET signal is greatly reduced (bottom right). (**C**) Sample data of image-based MT depolymerization assay from a single 96-well plate with the data points colored as labeled. The compound with a red circle is a putative inhibitor, whereas the compound with a purple circle is a putative activator. (**D**) Sample data of FRET assay from a single 96-well plate with the data colored as labeled. Two compounds with red circles are putative inhibitors whereas the compound with a purple circle is a putative activator. (**E**) Sample data of further optimized FRET assay from a single 384-well plate with the data as labeled. Two compounds with red circles are putative inhibitors, whereas the compound with a purple circle is a putative activator. Note that the difference in Y-axis scaling relative to (**D**) is due to the use of a spectral-based plate reader in the assay in (**E**). (**F**) Structure of the three hits identified from the small-scale screens. Created with Chemdraw.
