# Supplementary material for: MCAK Inhibitors Induce Aneuploidy in Triple-Negative Breast Cancer Models
Source: Cancers (Basel). 2023 Jun 23;15(13):3309. doi: 10.3390/cancers15133309 (PMC10340532; doi:10.3390/cancers15133309)
Supplement: Supplementary file 1 [file cancers-15-03309-s001.zip › cancers-2455380-supplementary.pdf]

Cell Line	Relative Expression	Reference	Tissue of Origin
MDA-MB-231	1.00	-	Breast
PTXR-231	1.00	In-house qPCR	Breast (MDA-MB-231 Derived)
MCF10A	0.38	†	Breast
HeLa	0.68	††	Cervix
U2OS	0.99	††	Bone
RPE-1	0.14	††	Retina

Table S1. MCAK expression in the cell lines used in this study normalized to expression in MDA-MB-231 cells. † Expression data obtained from the GSE10890 dataset: https://www.ncbi.nlm.nih.gov/geo/query/acc.cgi?acc=GSE10890. †† expression data obtained from the Human Protein Atlas: https://www.proteinatlas.org/ENSG00000142945-KIF2C/cell+line.

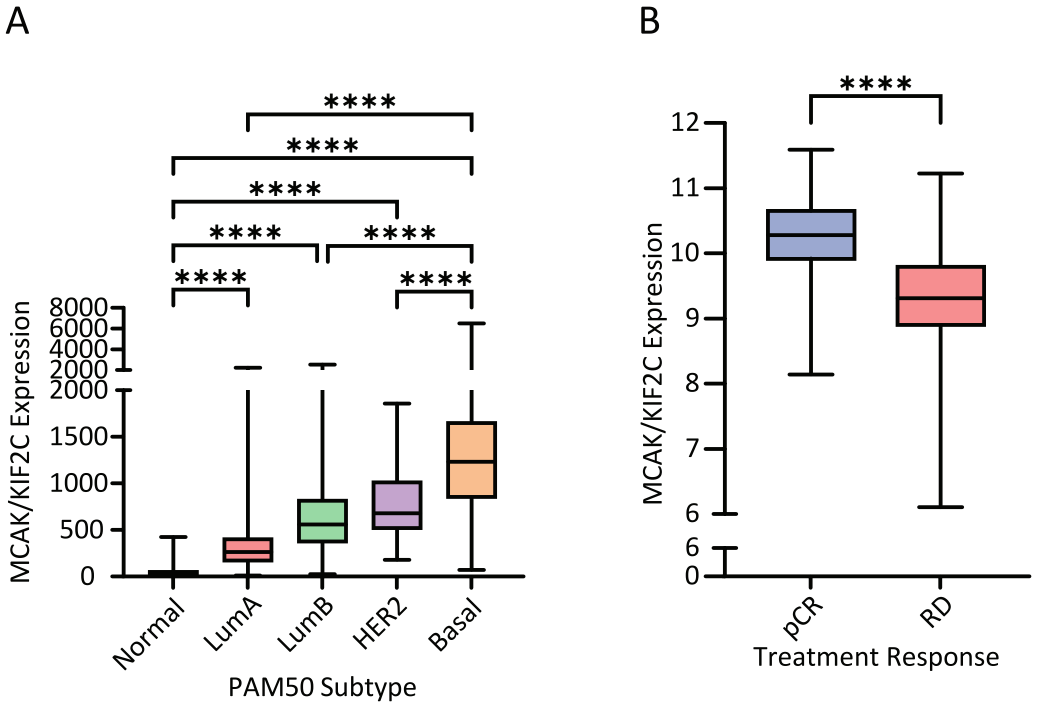

Figure S1. MCAK overexpression correlates with poor prognoses. (A) MCAK/KIF2C expression data from TCGA BC dataset from breast cancer tumors of different receptor status (Normal N = 112, LumA = 516, LumB = 261, HER2 = 80, Basal = 167) plotted as box-and-whisker plots where the box represents the upper and lower quartiles, and the whiskers represent the upper and lower ranges. ANOVA with Tukey's post hoc test for significance was used to compare groups. **** $p < 0.0001$. LumA=Luminal A; LumB=Luminal B; HER2=HER2-enriched. (B) MCAK/KIF2C expression data from GSE25066 dataset from breast tumors in which patients had either a pathological Complete Response (pCR; N = 196) or had Residual Disease (RD; N = 312). MCAK/KIF2C levels are plotted for each condition as a box-and-whisker plot. The Mann-Whitney test for significance was used to compare groups. **** $p<0.0001$.

A

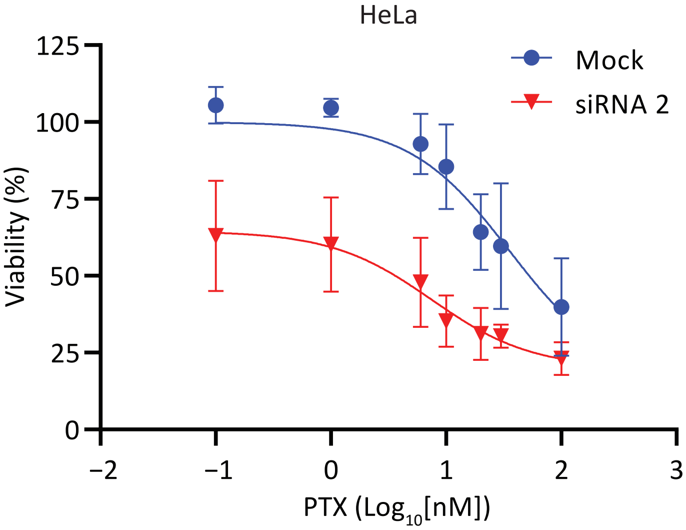

MCAK siRNA	-	2
PTX IC_{50} (nM)	35.38	7.50*

B

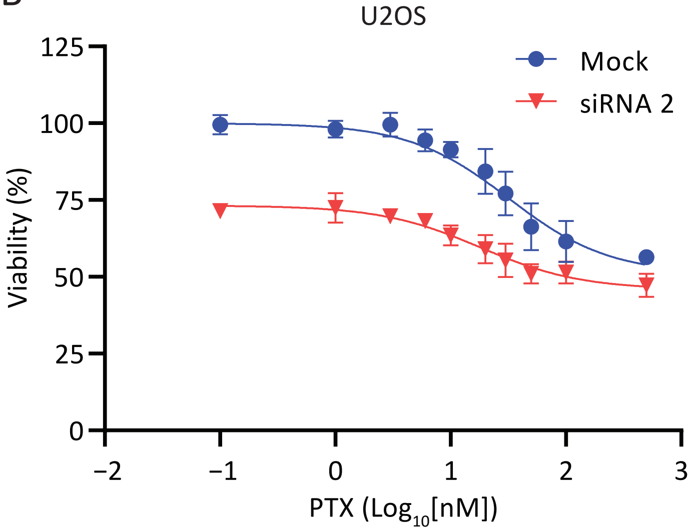

MCAK siRNA	-	2
PTX IC_{50} (nM)	31.99	18.16*

C

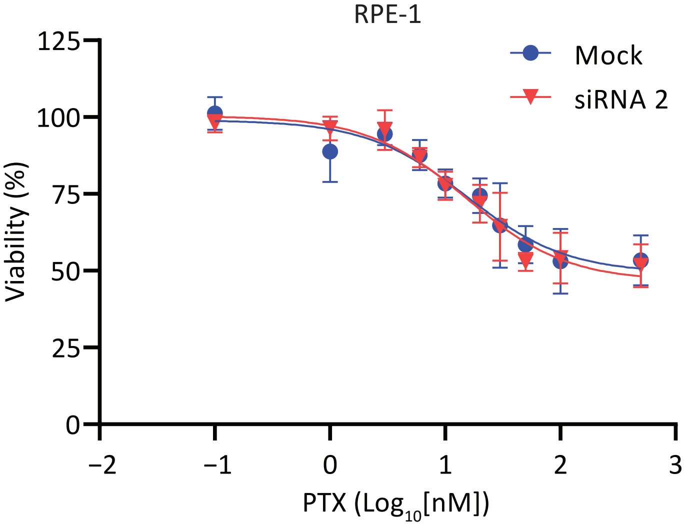

MCAK siRNA	-	2
PTX IC_{50} (nM)	16.35	14.68

D

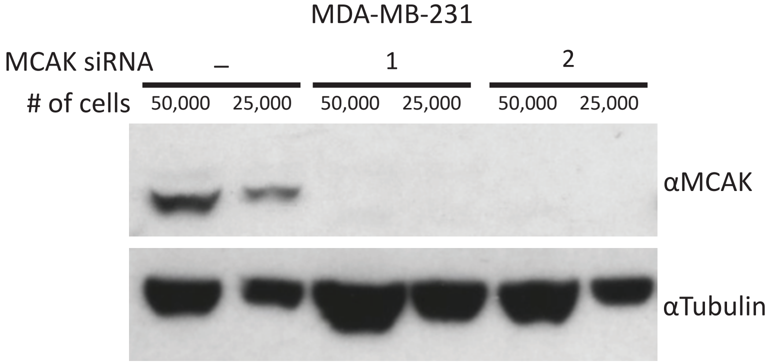

E

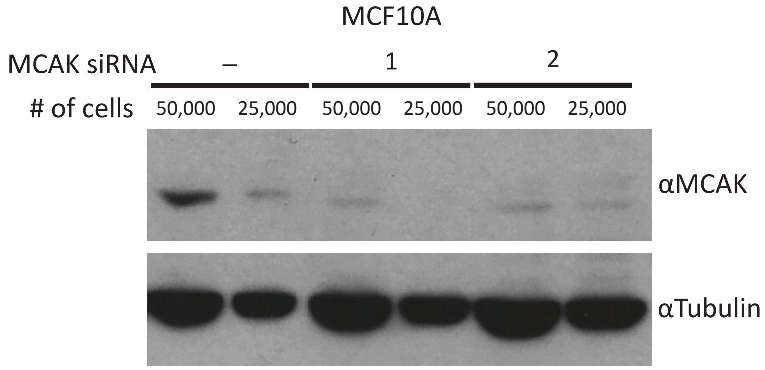

F

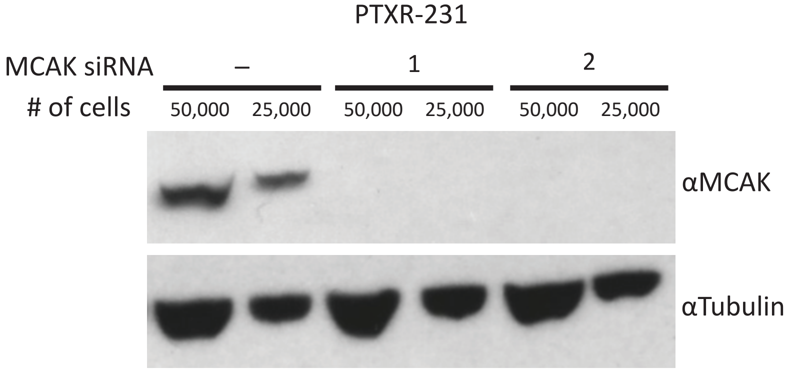

Figure S2. MCAK loss sensitizes cancer cells to taxanes. (A-C) Dose response curves and associated IC50 values of paclitaxel in HeLa (A), U2OS (B), and RPE-1 (C) cells from n = 6, 5, and 4 independent experiments respectively. * $p<0.05$. (D-F) Western blot of control Neg2 (-) or MCAK knockdown cells with two independent siRNAs (1 or 2) in either MDA-MB-231 (D), MCF10A (E) or PTXR-231 (F) cells. Blots were probed with anti-MCAK (top) or anti-alpha-tubulin (bottom).

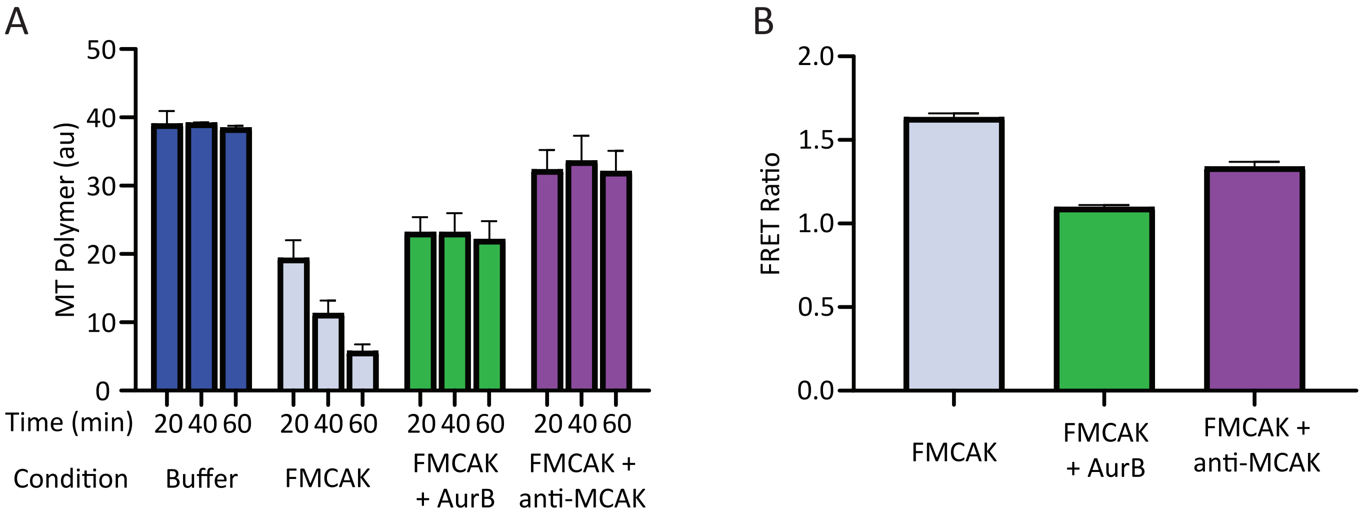

Figure S3. Controls for screening assays. (A) FIJI quantification of MT polymer amounts under the indicated conditions. Bars represent mean +/- SD of 10 replicates in a single 96-well plate and represents two independent experiments. (B) FRET ratios (mean +/- SD, N=10) of each control condition. Representative of two independent experiments.

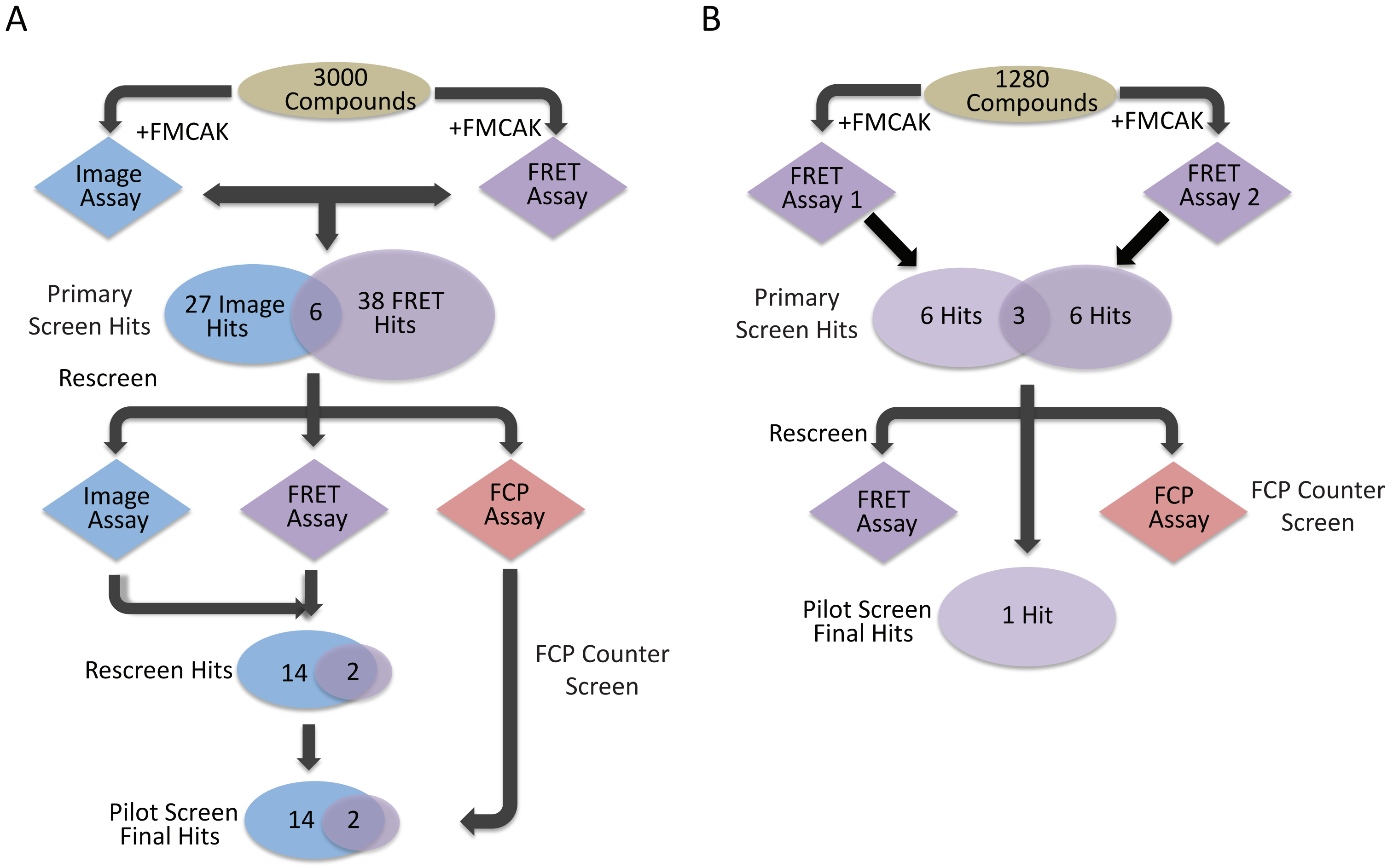

Figure S4. Summary of screens used to identify MCAK inhibitors. (A) Flowchart schematic of 3000-compound screen using both a MT depolymerization assay and a FRET-based assay. (B) Flowchart schematic of 1280-compound screen done in duplicate with the optimized FRET-based assay.

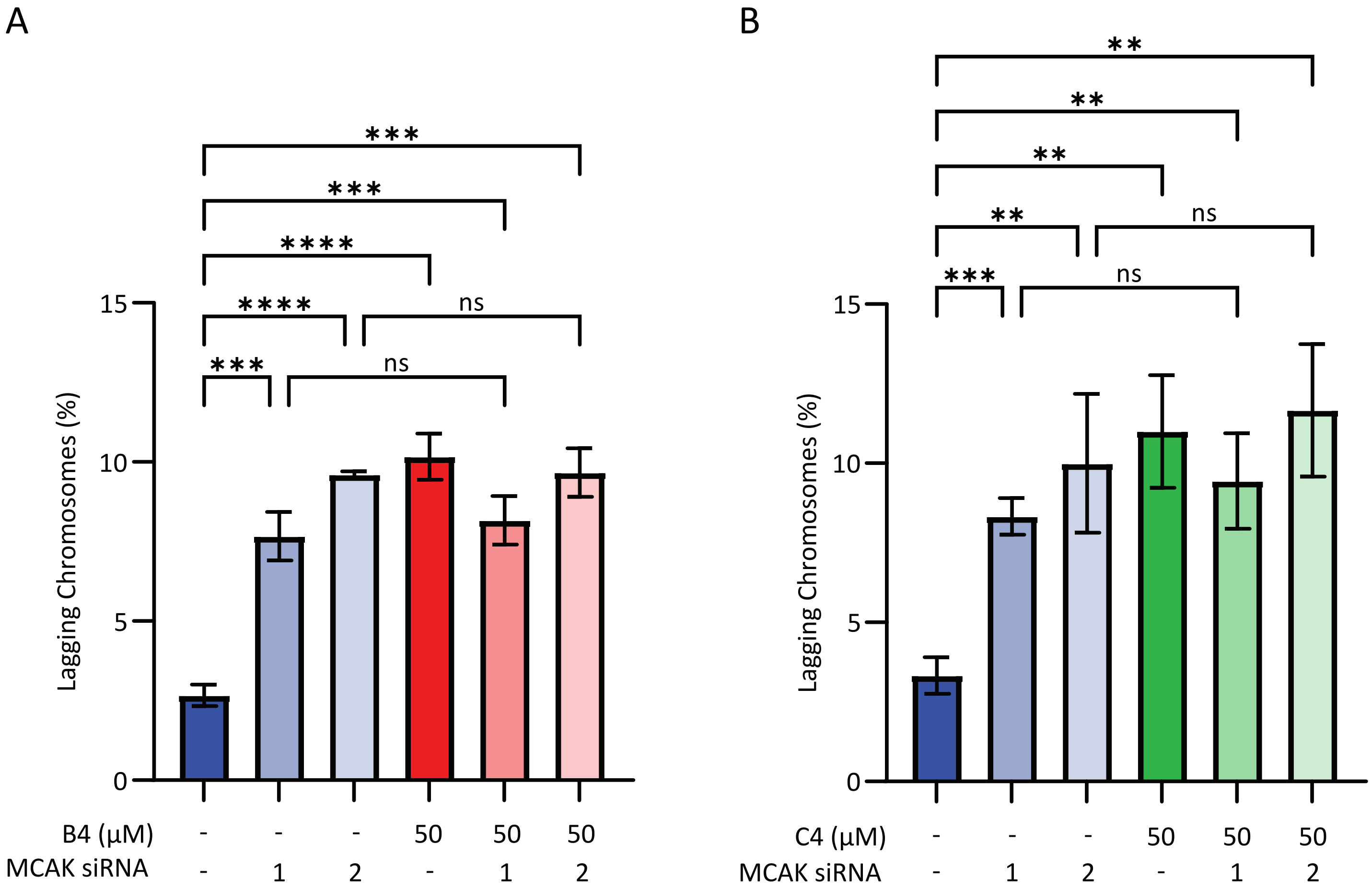

Figure S5. MCAK aneuploidy with knockdown in HeLa cells. (A, B) Percentage of HeLa cells in anaphase with lagging chromosomes. Cells were treated with control or MCAK siRNAs and DMSO, 50 μM B4 (A), or 50 μM C4 (B). Values represent the mean +/- SD Conditions were compared with a Student's t-test. ns = not significant, ** p<0.01, *** p<0.001, **** p<0.0001
